# Real-time forecasting of COVID-19 bed occupancy in wards and Intensive Care Units

**DOI:** 10.1007/s10729-021-09553-5

**Published:** 2021-03-25

**Authors:** Stef Baas, Sander Dijkstra, Aleida Braaksma, Plom van Rooij, Fieke J. Snijders, Lars Tiemessen, Richard J. Boucherie

**Affiliations:** 1 Center for Healthcare Operations Improvement and Research (CHOIR), University of Twente, Enschede, The Netherlands; 2 Elisabeth-TweeSteden Ziekenhuis, Tilburg, The Netherlands; 3 Leiden University Medical Centre, Leiden, The Netherlands; 4 Rijnstate, Arnhem, The Netherlands

**Keywords:** COVID-19, Forecast, Bed occupancy, Network of infinite server queues, Richards’ curve, Kaplan-Meier estimator

## Abstract

This paper presents a mathematical model that provides a real-time forecast of the number of COVID-19 patients admitted to the ward and the Intensive Care Unit (ICU) of a hospital based on the predicted inflow of patients, their Length of Stay (LoS) in both the ward and the ICU as well as transfer of patients between the ward and the ICU. The data required for this forecast is obtained directly from the hospital’s data warehouse. The resulting algorithm is tested on data from the first COVID-19 peak in the Netherlands, showing that the forecast is very accurate. The forecast may be visualised in real-time in the hospital’s control centre and is used in several Dutch hospitals during the second COVID-19 peak.

## Highlights

- A network of infinite server queues driven by a Poisson Arrival Location Model is developed to model COVID-19 ward and Intensive Care Unit (ICU) occupancy forecasts.
- Data-driven forecasts are generated, fully based on data readily available from the hospital’s data warehouse.
- Forecasts are very accurate. In particular, forecasts of the maximum occupancy in the ward and the ICU three days ahead are very close to their realisation.
- These forecasts are currently being used in four Dutch hospitals during the second COVID-19 peak the Netherlands is facing.

## Introduction

The COVID-19 pandemic impacts people’s health, jobs and well-being and puts an enormous strain on healthcare resources. This is also the case in the Netherlands, where hospital resources are under pressure due to the large number of hospitalised COVID-19 patients, which moreover results in reduction of resources available for non-COVID-19 patients [26]. An accurate forecast of the number of COVID-19 patients being hospitalised supports allocation of the resources required for treatment of both COVID-19 and non-COVID-19 patients. This paper presents a mathematical model that provides a real-time forecast of the number of COVID-19 patients admitted to the ward and the Intensive Care Unit (ICU) of a hospital based on the predicted inflow of patients, their Length of Stay (LoS) in both the ward and the ICU as well as transfer of patients between the ward and the ICU. The data required for this forecast is obtained directly from the hospital’s data warehouse and the forecast is available to the hospital in real-time.

Forecasting the number of hospitalised COVID-19 patients is required to determine the resource allocation to COVID-19 and non-COVID-19 patients. A COVID-19 patient’s medical condition may change rapidly and unexpectedly [21]. As a consequence, it is not possible to accurately forecast the COVID-19 patients’ resource requirements many days ahead of time [12]. We therefore focus on forecasting the number of hospitalised COVID-19 patients one to five days ahead of time. In particular, we are interested in forecasting the mean number of patients present and the risk of bed shortage, expressed as the probability that the maximum number of COVID-19 patients in the ward and ICU exceeds a pre-specified safety level. Several studies have demonstrated the Erlang loss model (or *M*/*G*/*c*/*c* queue) to be suitable for dimensioning of isolated wards (e.g., [5, 29]) and ICUs (e.g., [2, 6]). Such models typically assume constant arrival rates and LoS distributions. In our context, however, the LoSs of patients may vary over time due to improved treatment, arrivals of patients are non-stationary, and patients may transfer between ward and ICU. In such settings with time-varying load, a network of Erlang loss queues can well be approximated by a network of infinite server queues [1, 19], either using a Pointwise Stationary Approximation or a Modified Offered Load Approximation. The advantage of this approximation is that it allows explicit evaluation of performance measures. Hence, for our case, the most suitable model is a network of two infinite server queues with time-varying Poisson arrivals and generally distributed time-varying LoSs. We build upon the results for networks of infinite server queues as presented in [4, 19, 27] to allow for time-varying arrival rates and patient-specific time-varying LoS distributions.

With data on COVID-19 patients becoming more and more available, prediction of the infection rate a few days ahead of time [10, 31], and of the LoS [22] is possible. Predictions of the number of hospitalised COVID-19 patients based on regression methods are, e.g., reported in [7, 8, 18]. The LoS distribution of COVID-19 ICU patients in the United Kingdom is fitted to probability distributions in [28]. Our model combines such predictions to forecast the number of patients residing in the COVID-19 ward and ICU. We have chosen to predict the arrival rates and LoS directly from the hospital’s data warehouse as external data does not represent the case mix of the hospital. Our method requires a complete set of time stamps for patient admissions, transfers and discharges that is made available by the participating hospitals. We use a Richards’ curve [25] to predict the arrival rates. The Richards’ curve is a growth model that can be used to describe the cumulative total number of hospitalised COVID-19 patients, i.e., ward and ICU combined. The Richards’ curve was introduced to describe processes in biological systems, but has recently gained popularity in predicting the outbreak of diseases. For instance, the Richards’ curve has been successfully applied to predict the daily number of new COVID-19 infection cases in (provinces of) China and countries in Europe [16, 31]. We estimate the distribution of the LoS in both the COVID-19 ward and ICU using a Kaplan-Meier estimator [13]. Analytical evaluation of the exact expressions for the rates of the time-dependent distribution of bed occupancy is prohibitive. Therefore, in our forecasting algorithm we use a Monte-Carlo method to sample from the LoS distributions. We forecast the mean number of patients present and the risk of bed shortage, expressed as the maximum number of COVID-19 patients at the ward and ICU at a number of subsequent days, including the corresponding prediction intervals. In particular, we sample the patient trajectories in the Poisson Arrival Location Model [19] that determines the queue occupancy in our network of infinite server queues. As such, our method may be viewed as a data-driven approach that forecasts the number of hospitalised COVID-19 patients based on estimated arrival rates and LoSs, justified by an underlying queueing model. The algorithm is implemented in R version 3.6.3 and first tested on data from the first COVID-19 peak for four hospitals in the Netherlands. The forecast is found to be very accurate.

Forecasting the number of hospitalised COVID-19 patients is difficult [12]. As stated above, various approaches exist based on regression methods [7, 8, 10, 18, 31], and on estimation of the LoS [22, 28]. The key to the accuracy of our forecast is that admissions are predicted according to a Richards’ curve, which is proven to be an accurate predictor for the number of COVID-19 infections [16, 31], while the joint effect of the arrival rates and the LoS is taken into account via the underlying network of infinite server queues. This enables evaluation of the future evolution of bed occupancy via the trajectories of the Poisson Arrival Location Model, taking into account patient admissions, transfers and discharges. The algorithm currently runs in four hospitals to forecast the number of hospitalised COVID-19 patients during the second peak the Netherlands is currently facing.

This paper is organised as follows. Section 2 presents our modelling assumptions and the network of two infinite server queues that we propose to forecast the number of hospitalised COVID-19 patients. Section 3 describes the statistical forecasting approach used in our method, and Section 4 presents forecasts using data of the first COVID-19 peak in the Netherlands. Occupancy is most easily forecast in a large hospital with a homogeneous patient mix. In Section 4, we present forecasting results for a medium-sized, academic hospital as well as for a number of larger hospitals, and reflect on the accuracy of our forecasts. Finally, Section 5 wraps up the paper with concluding remarks and our aims for further research: extending our model to a regional model including patient transfers between hospitals.

## Model

**Fig. 1:**
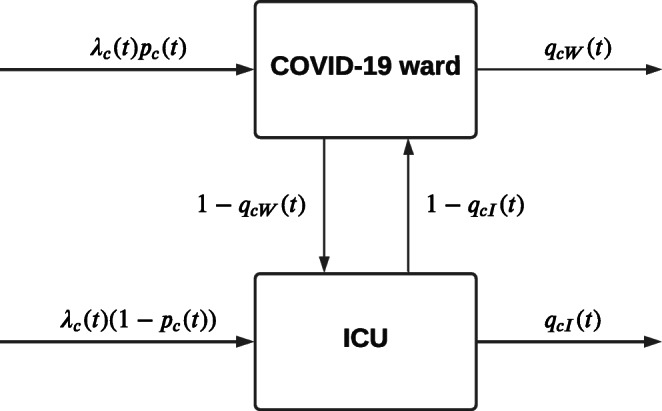
Patient flows

Upon COVID-19 infection, some patients develop mild or no symptoms, whereas others develop symptoms that require hospitalisation at either the ward or Intensive Care Unit (ICU) depending on, e.g., the need for artificial respiration [11, 21]. While hospitalised, a patient’s condition may worsen, resulting in a transfer from the ward to the ICU or death, or a patient may recover, resulting in a transfer from ICU to ward or a discharge from the ward. Note that discharges from the ICU other than death are rare and mainly correspond to transfer of the patient to another hospital. Some patients admitted to the ward have treatment restrictions that prohibit transfer from ward to ICU. As COVID-19 is a relatively new disease, the evolution of a patient’s condition and the effect of treatment are still under investigation. A patient’s Length of Stay (LoS) at the ward or ICU may depend on patient characteristics such as age, gender, BMI and treatment restrictions, may differ considerably between hospitals due to different treatment protocols or differences in case mix, and may also change over time due to, e.g., improved treatment [3, 21, 22]. Therefore, we estimate the distribution of the LoS and the probability of patient transfers between ward and ICU from the data on COVID-19 patients in the hospital’s data warehouse. Arrivals of new patients are influenced by the number of infections in the hospital’s region, and also by the characteristics of the hospital, e.g., more severely ill patients will be admitted to university medical centres, whereas less ill patients may be treated in local hospitals and may be transferred if their condition worsens. Therefore, we predict the rate of admittance of COVID-19 patients from the hospital’s data warehouse record of earlier admissions. Figure 1 depicts the flow of patients in the COVID-19 ward and ICU.

We now develop a network of two infinite server queues with multiple patient-types, time-dependent arrival process, and general and time-dependent LoS that records the number of hospitalised patients. Consider a hospital that admits COVID-19 patients to its ward and ICU. The arrival rate of patients is determined by the number infected in the hospital’s region and a consequence of the infection rate in that region. In agreement with arrivals to Emergency Departments, the arrival process may be modelled as a Poisson process with time-dependent rate [30]. Consider a set *C* of patient characteristics, including, e.g., age, gender, BMI, and treatment restrictions. Let patients with characteristics *c* ∈ *C* arrive with rate *λ*_*c*_(*t*), where *t* denotes time. A fraction *p*_*c*_(*t*) of these patients is admitted to the ward, the other patients are admitted to the ICU. Let random variables *L*_*cW*_(*t*) and *L*_*cI*_(*t*) denote the LoS of a patient with characteristics *c* ∈ *C* admitted or transferred to the ward and ICU, respectively, at time *t*. Let *q*_*cW*_(*t*), *q*_*cI*_(*t*) denote the probability that a patient admitted or transferred to the ward or ICU is discharged or dies, at time *t*. Then 1 − *q*_*cW*_(*t*) and 1 − *q*_*cI*_(*t*) are the probabilities of transfer from ward to ICU and vice versa upon completion of the LoS. We assume that patients do not interfere with each other, hence that all random variables related to patients’ arrival, transfer, and LoS are independent.

Characteristics of a patient’s LoS and transfer probabilities are related to the time the patient is admitted. As a consequence, we may model the system as a network of two infinite server queues with multiple job-types, time-varying arrival rates and general LoS distribution. To this end, let *N*_*cW*_(*t*) and *N*_*cI*_(*t*) record the number of patients with characteristics *c* at time *t* in the ward and ICU, respectively. These random variables have a time-dependent Poisson distribution, for *n*_*cW*_,*n*_*cI*_ = 0,1,2…:

1 $$ \begin{array}{@{}rcl@{}} \mathbb{P}[N_{cW}(t)&=&n_{cW},N_{cI}(t)=n_{cI}]\\ &=& \frac{\rho_{cW}(t)^{n_{cW}}}{{n_{cW}}!}\frac{\rho_{cI}(t)^{n_{cI}}}{{n_{cI}}!} \mathrm{e}^{-(\rho_{cW}(t)+\rho_{cI}(t))}, \end{array} $$

where the means *ρ*_*cW*_(*t*), *ρ*_*cI*_(*t*) are, in closed-form, determined by *λ*_*c*_(*t*), *p*_*c*_(*t*), *L*_*cW*_(*t*), *L*_*cI*_(*t*), *q*_*cW*_(*t*) and *q*_*cI*_(*t*), via the Poisson Arrival Location Model, as integrals over a location function, see [19, Theorem 2.1]. If the LoSs are exponentially distributed with rates *μ*_*cW*_ and *μ*_*cI*_ at the ward and ICU, then the means *ρ*_*cW*_(*t*), *ρ*_*cI*_(*t*) may be obtained from

$$ \begin{array}{@{}rcl@{}} \frac{1}{\mu_{cW}} \frac{d \rho_{cW}(t)}{dt} &=& \lambda_{c}(t)p_{c}(t) + \rho_{cI}(t) (1-q_{cI}(t)) - \rho_{cW}(t), \\ \frac{1}{\mu_{cI}} \frac{d \rho_{cI}(t)}{dt} &=& \lambda_{c}(t)(1-p_{c}(t)) + \rho_{cW}(t) (1-q_{cW}(t)) - \rho_{cI}(t), \end{array} $$

with initial conditions

2 $$ \rho_{cW}(0)=\rho_{cW}^{*}, \quad \rho_{cI}(0)=\rho_{cI}^{*},  $$

that reflect the current number of hospitalised patients in the ward and ICU at the starting time 0 of our forecasting period [4]. If the LoS has a general distribution not depending on the arrival time of the patients, and transfer probabilities do not depend on time, the means *ρ*_*cW*_(*t*), *ρ*_*cI*_(*t*) are obtained as

3 $$ \begin{array}{@{}rcl@{}} \rho_{cW}(t) &=& \mathbb{E}\left[ {\int}_{t-L_{cW}}^{t} \lambda^{+}_{cW}(u)du \right] , \end{array} $$

4 $$ \begin{array}{@{}rcl@{}} \rho_{cI}(t) &=& \mathbb{E}\left[ {\int}_{t-L_{cI}}^{t} \lambda^{+}_{cI}(u)du \right], \end{array} $$

with

$$ \begin{array}{@{}rcl@{}} \lambda^{+}_{cW}(t)&=& \lambda_{c}(t)p_{c}(t) +\mathbb{E}\left[ \lambda_{cI}^{+}(t-L_{cI})\right](1-q_{cI}) , \\ \lambda^{+}_{cI}(t)&=& \lambda_{c}(t)(1-p_{c}(t)) +\mathbb{E}\left[ \lambda_{cW}^{+}(t - L_{cW})\right](1 - q_{cW}), \end{array} $$

see [19, Theorem 1.2]. In our network of ward and ICU with time-varying LoS and transfer probabilities, the expressions for *ρ*_*cW*_(*t*), *ρ*_*cI*_(*t*) are more involved. Moreover, the arrival rates, LoS distributions and transfer probabilities are not available in closed form, which prohibits evaluation of the expectations in (3), (4). Therefore, we do not provide an explicit expression for these means in the general case. In Section 3 we provide an algorithm that predicts the arrival rates and estimates the LoSs and transfer probabilities from the hospital’s data warehouse. Subsequently, we use these system parameters to sample the patient trajectories of the Poisson Arrival Location Model resulting in a forecast of *ρ*_*cW*_(*t*), *ρ*_*cI*_(*t*) given the initial Poisson distribution of the number of patients as reflected by the initial condition (2) and the residual LoS of these patients.

Let $N_{W}(t)={\sum }_{c}N_{cW}(t)$ and $N_{I}(t)={\sum }_{c}N_{cI}(t)$ record the total number of patients in the ward and ICU, respectively, at time *t*. The distribution of *N*_*W*_(*t*) and *N*_*I*_(*t*) is now readily obtained. These random variables have a time-dependent Poisson distribution, for *n*_*W*_,*n*_*I*_ = 0,1,2…:

5 $$  \mathbb{P}[N_{W}(t)=n_{W},N_{I}(t)=n_{I}] = \frac{\rho_{W}(t)^{n_{W}}}{{n_{W}}!}\frac{\rho_{I}(t)^{n_{I}}}{{n_{I}}!} \mathrm{e}^{-(\rho_{W}(t)+\rho_{I}(t))}, $$

with

$$ \rho_{W}(t)=\sum\limits_{c \in C}\rho_{cW}(t), \quad \rho_{I}(t)=\sum\limits_{c \in C}\rho_{cI}(t). $$

Observe from (5) that at each time *t* the random variables *N*_*W*_(*t*), *N*_*I*_(*t*) are independent. However, for different time points, say *t*_1_ and *t*_2_, the random variables *N*_*W*_(*t*_1_), *N*_*I*_(*t*_2_) are correlated, see [19, Theorem 2.2].

The Poisson distributions for the number of hospitalised patients (1), (5) allow us to evaluate various performance measures. Let **L**(*s*) be a tuple that contains information on the number of patients in the ward and ICU, their patient characteristics *c*, and the realised LoSs (up to time *s*) of patients residing the ward and ICU at time *s*. First, we forecast the occupancy at the ward and ICU at time *s* + *t* given the LoSs of the residing patients at time *s*:

6 $$ \begin{array}{@{}rcl@{}} && \mathbb{E}[N_{W}(s+t) | \textbf{L}(s)= {\boldsymbol\ell}], \\ &&\mathbb{E}[N_{I}(s+t) | \textbf{L}(s)= {\boldsymbol\ell}]. \end{array} $$

Furthermore, for a confidence level *α* ∈ [0,1], we are interested in the quantiles

7 $$ \begin{array}{@{}rcl@{}} l_{\alpha W}(s+t), r_{\alpha W}(s+t), l_{\alpha I}(s+t), r_{\alpha I}(s+t) \end{array} $$

such that

8 $$ \begin{array}{@{}rcl@{}} &&\mathbb{P}\Big[N_{W}(s+t)\in[l_{\alpha W}(s+t),r_{\alpha W}(s+t)) | \textbf{L}(s)= {\boldsymbol\ell}\Big] \geq \alpha ,\\ &&\mathbb{P}\Big[N_{I}(s+t)\in[l_{\alpha I}(s+t),r_{\alpha I}(s+t)) | \textbf{L}(s)= {\boldsymbol\ell}\Big] \geq \alpha . \end{array} $$

These quantiles give level *α* prediction intervals for the occupancy of the ward and ICU at time *s* + *t*, conditional on all LoS information of the currently residing patients. Second, we forecast the expected maximum occupancy at the ward and ICU from time *s* up to time *s* + *t* given the LoS of the residing residents at time *s*:

9 $$ \begin{array}{@{}rcl@{}} &&\mathbb{E}\left[\max_{u\in[s,s+t]}N_{W}(u) | \textbf{L}(s)= {\boldsymbol\ell}\right] ,\\ &&\mathbb{E}\left[\max_{u\in[s,s+t]}N_{I}(u) | \textbf{L}(s)= {\boldsymbol\ell}\right]. \end{array} $$

For some confidence level *α* ∈ [0,1], we are interested in the quantiles

10 $$ l_{\alpha mW}(s+t), r_{\alpha mW}(s+t), l_{\alpha mI}(s+t), r_{\alpha mI}(s+t), $$

such that

11 $$ \begin{array}{@{}rcl@{}} & &\mathbb{P}\left[\max_{u\in[s,s+t]}N_{W}(u)\in\left[l_{\alpha mW}(s+t), r_{\alpha mW}(s+t)\right) | \textbf{L}(s)= {\boldsymbol\ell}\right] \geq \alpha, \\ & & \mathbb{P}\left[\max_{u\in[s,s+t]}N_{I}(u)\in\left[l_{\alpha mI}(s+t), r_{\alpha m I}(s+t)\right) | \textbf{L}(s)= {\boldsymbol\ell}\right] \geq \alpha. \end{array} $$

These quantiles give level *α* prediction intervals for the maximum occupancy of the ward and ICU during the interval [*s*,*s* + *t*], conditional on all LoS information of the currently residing patients. This is of particular interest for the decision to accept new COVID-19 patients. Other performance measures, including the mixture of patients in the ward and ICU at each time *t*, may be obtained from (1).

## On-line forecasting method

This section provides a procedure to predict the arrival rates (Section 3.1) and estimate the LoS distribution and transfer probabilities (Section 3.2) from the hospital’s data warehouse. In Section 3.3 we use these system parameters to sample the patient trajectories of the Poisson Arrival Location Model resulting in forecasts of the daily occupancy and the maximum occupancy, including their prediction intervals.

### Richards’ curve to predict the arrival rate

Our forecasting method requires *λ*(*t*), the expected number of arrivals of patients to the hospital at time *t*. In accordance with literature [16] the cumulative rate follows a 5-parameter Richards’ curve:

12 $$ {\Lambda}(t) ={\int}_{-\infty}^{t}\lambda(s)ds = \frac{R-L}{[1+ \delta\exp(-k (t-t_{0}))]^{1/\delta}} + L,  $$

where all parameters *R*,*δ*,*k*,*t*_0_ are positive, resulting in an S-shaped growth curve for the expected number of arrivals up to time *t*. The parameters have the following interpretations: *R* represents the total number of arrivals (indeed $\lim _{t\rightarrow \infty }{\Lambda }(t)=R$), *k* is a scale parameter related to the growth rate, *t*_0_ determines the offset along the *t*-axis, *δ* is a shape parameter that introduces asymmetry and *L* is a left asymptote of Λ(*t*). While the proposed method for estimating the arrival process is essentially data driven, it also has links to epidemiological models. For instance, if *δ* = 1, the resulting growth curve is the logistic growth curve that describes the fraction of infected people in a Susceptible-Infected-Susceptible (SIS) compartmental model [9].

The Richards’ curve is fitted on the cumulative number of arrivals using the R package FlexParamCurve, version1.5-5 [23] via the following procedure. Our goal is to forecast the occupancy of the ward and ICU on a daily basis. To this end, the number of arrivals of COVID-19 patients is determined for each day from the hospital’s data warehouse. The parameters of the Richards’ curve are estimated by minimising the sum of squared errors between the cumulative number of arrivals on each day and the expression in the right-hand side of (12) at that day using the Levenberg-Marquardt algorithm, i.e. the exact same procedure as in [31]. The Levenberg-Marquardt algorithm is implemented in the R package minpack.lm, version 1.2-1 [20].

For some sets of arrival data, the nonlinear least squares method fails to converge due to over-parameterisation. In this case, the procedure first fixes the parameter *L* to 0 (leading to a 4-parameter Richards’ curve). If this also does not lead to convergence, the parameter *δ* is fixed to 1, in which case the procedure fits a logistic growth curve on the arrival data. This procedure results in an estimate $\tilde {\Lambda }(d)$ for the expected cumulative number of arrived COVID-19 patients on any given day *d*. This daily estimate is then linearly interpolated to generate a cumulative arrival intensity $\hat {\Lambda }(t)$ which can be evaluated at each time point *t*. Let $\hat {p}$ denote the empirically estimated probability *p*_*c*_(*t*) that a patient with characteristics *c* is admitted to the ward at time *t*, which is assumed stationary. Let $\hat {f}_{c}(t)$ be the empirically estimated fraction of patients with characteristics *c* that arrive directly to the hospital at time *t*. The cumulative arrival rate $\hat {\Lambda }_{c}(t)$ of patients with characteristics *c* arriving directly to the hospital is then estimated as $\hat {\Lambda }_{c}(t) = \hat {f}_{c}(t)\hat {\Lambda }(t)$ for all *t*, and the cumulative arrival rates to the ward and ICU are estimated as $\hat {p}_{c}\hat {f}_{c}(t)\hat {\Lambda }(t)$ and $(1-\hat {p}_{c})\hat {f}_{c}(t)\hat {\Lambda }(t)$ for all *t*.

### Kaplan-Meier estimation of the LoS distribution and transfer probabilities

Our forecasting method requires the LoS distribution *F*_*cW*_ at the ward and *F*_*cI*_ at the ICU for all patient characteristics *c*. We use the Kaplan-Meier estimator [13] for the survival function that takes right-censored observations into account, which occur when a patient is still at the respective department or when a patient is transferred to another hospital. Our goal is to forecast the occupancy at the ward and ICU on a daily basis. The estimated LoS distribution gives the probability that a patient is at the department at most a certain number of days. Let *e*_*cW*_(*v*), resp. *e*_*cI*_(*v*), denote the number of patients with characteristics *c* at the ward, resp. ICU, with a realised LoS equal to *v*. Let *n*_*cW*_(*v*), resp. *n*_*cI*_(*v*) denote the number of patients with characteristics *c* at the ward, resp. ICU, with a LoS at least equal to *v*. These numbers are aggregated over arrival times at the department to increase the sample size for the Kaplan-Meier estimates. The Kaplan-Meier estimates for the LoS distribution at the ward and ICU are then given by [13]:

$$ \begin{array}{@{}rcl@{}} &&\hat{F}_{cW}(\ell) = 1- \prod\limits_{v=1}^{\ell}\left( 1-\frac{e_{cW}(v)}{n_{cW}(v)}\right),\\ && \hat{F}_{cI}(\ell) = 1- \prod\limits_{v=1}^{\ell}\left( 1-\frac{e_{cI}(v)}{n_{cI}(v)}\right). \end{array} $$

The Kaplan-Meier estimates are calculated using the R package survminer, version 0.4-6 [14].

Our method also requires an estimate of the probabilities *q*_*cW*_(*ℓ*) (*q*_*cI*_(*ℓ*)) that a patient with characteristics *c* and LoS *ℓ* at the ward (ICU) is discharged or dies. Let $\hat {F}_{cIW}$ denote the empirical probability that a patient with characteristics *c* is transferred from the ICU to the ward after at most a LoS of *ℓ* days at the ICU, and let $\hat {F}_{cWI}(\ell )$ be defined similarly for transfers from ward to ICU. The probabilities *q*_*cW*_(*ℓ*) and *q*_*cI*_(*ℓ*) are estimated as

$$ \begin{array}{@{}rcl@{}} &&\hat{q}_{cW}(\ell) = 1-\frac{\hat{F}_{cWI}(\ell) - \hat{F}_{cWI}(\ell-1)}{\hat{F}_{cW}(\ell)-\hat{F}_{cW}(\ell-1)},\\&&\hat{q}_{cI}(\ell) = 1-\frac{\hat{F}_{cIW}(\ell) - \hat{F}_{cIW}(\ell-1)}{\hat{F}_{cI}(\ell)-\hat{F}_{cI}(\ell-1)}. \end{array} $$

### Generation of the Poisson Arrival Location Model and forecasting ward and ICU bed occupancy

This section presents our method to sample the patient trajectories of the Poisson Arrival Location Model (PALM) resulting in, for instance, forecasts of the conditional means shown in (6). Our method simulates the PALM using Monte Carlo sampling of arrivals, transfers and departures of patients over the forecasting period [*s*,*s* + *t*]. We restrict the trajectories to:

1. patients admitted to the ward that leave the hospital from the ward,
2. patients admitted to the ICU that leave the hospital from the ICU,
3. patients admitted to the ICU, then transferred to the ward and leave the hospital from the ward,
4. patients admitted to the ward, then transferred to the ICU and leave the hospital from the ICU.

As a consequence, in our simulation method a patient may visit at most two departments. This restriction is introduced to reduce computational complexity as it avoids a large number of possible patient trajectories. The restriction has a minor effect on our results, as data shows that multiple transfers are very rare during our forecasting horizon of at most one week. In the description of our method below, we identify the patient characteristics *c* with the trajectories (i.e. *c* ∈{1,2,3,4}). Note that randomly assigning patients to these trajectories is probabilistically equivalent to random selection of transfer or discharge/death upon completion of the LoS at a department, see, e.g., [15, p. 64].

All parameters required for our sampling method are obtained from the hospital’s data warehouse as presented in Sections 3.1 and 3.2.

For each time *u* in the forecasting period [*s*,*s* + *t*], arrivals of new patients with characteristics *c* are sampled according to a non-homogeneous Poisson process with cumulative rate equal to $\hat {p}_{c}f_{c}(u)\hat {\Lambda }(u)$ for the ward and $(1-\hat {p}_{c})f_{c}(u)\hat {\Lambda }(u)$ for the ICU, where the cumulative total arrival rate $\hat {\Lambda }(u)$ is extrapolated from the Richards’ curve based on the hospital’s data up to the start of the forecasting period at time *s*. The sampling procedure is executed until the next arrival time exceeds *s* + *t*. Sampling of inter-arrival times is executed by inverse transformation sampling based on the cumulative arrival rates (see, e.g., [24, p. 312]).

Departure times are generated upon the arrival/transfer of patients and is done by inverse sampling with replacement under the estimated empirical LoS distributions for that combination of patient characteristics and department. These LoS distributions are estimated on all LoS data obtained before the start of the forecasting period at time *s*. Note that for this specification of patient characteristics, the probability $\hat {q}_{cI}$ (resp. HCode $\hat {q}_{cW}$) of transfer from the ICU to the ward (resp. ward to ICU) is either always equal to zero or equal to one, depending on the characteristics (trajectory) *c* for that patient.

For patients residing at the hospital at time *s*, the trajectory is first sampled based on the patient’s current realised LoS *ℓ*. Conditional sampling is based on the ratio between Kaplan-Meier Survival function estimates of general patients residing in the current department and the Kaplan-Meier Survival function estimate of patients being transferred to the other department. For instance, for a patient currently residing at the ICU with current LoS equal to *ℓ*_0_, the probability that this patient follows trajectory 3 is equal to

$$ \frac{\hat{p}_{IW}(1-\hat{F}_{3IW}(\ell_{0}))}{1-\hat{F}_{I}(\ell_{0})}, $$

where $\hat {F}_{I}$ is a Kaplan-Meier estimate of the LoS distribution at the ICU for a general patient, i.e., estimated on all LoS data at the ICU and $\hat {p}_{IW}$ is the empirical (unconditional) probability of going from the ICU to the ward.

The LoS for these currently residing patients is then sampled from the empirical conditional LoS distribution for the sampled patient’s type, where conditioning is based on the already realised LoS. For example, if the patient follows trajectory 3 and has a current LoS of *ℓ*_0_ days at the ICU, the total LoS of this patient at the ICU is sampled from the cumulative distribution:

$$ \hat{F}_{3IW}(\ell|\ell_{0}) = \frac{\hat{F}_{3IW}(\ell)-\hat{F}_{3IW}(\ell_{0})}{1-\hat{F}_{3IW}(\ell_{0})}\quad \quad\forall \ell\geq \ell_{0}. $$

In order to keep track of the PALM, *N*_*I*_(*s*) and *N*_*W*_(*s*) are first determined. Then, at each simulated arrival, transfer or departure before time *s* + *t*, the counters are updated according to the nature of the event. This results in a registered occupancy for both departments at each day and time point in the forecasting period [*s*,*s* + *t*]. The realisation and forecast of the occupancy at a given day at a department is now calculated as the number of patients at the department at the start of that day (i.e., at that day at midnight, but another time point may easily be incorporated in our method).

The simulation procedure is repeated 1,000 times in order to accurately estimate the statistics mentioned at the end of Section 2. In order to estimate the expected values at day *s* + *t* in (6), the average of the number of patients on day *s* + *t* at both departments is taken over all simulations runs. Next, in order to estimate the boundaries in (7) for a given day *s* + *t*, the respective empirical quantiles are taken over the simulated occupancy on that day for both departments. Furthermore, in order to determine the expressions in (9) and (10), the maximum occupancy is determined for both departments from the forecast day (time *s*) until the end of the forecasting horizon (time *s* + *t*). Then, to determine (9) and (10), the empirical mean and quantiles are determined.

## COVID-19 bed occupancy: case studies and evaluation

This section presents the results of our forecasting method detailed in Section 3 for four Dutch hospitals. Section 4.1 presents results for the Leiden University Medical Centre (LUMC), a medium-sized academic hospital. Subsequently, we present results for the larger general hospitals HagaZiekenhuis (Section 4.2), Rijnstate (Section 4.3), and Elisabeth-TweeSteden Ziekenhuis (Section 4.4). In Section 4.5, we compare these forecasts, elaborate on the quality of our forecasts and provide a statistical evaluation of our forecasting method.

**Fig. 2:**
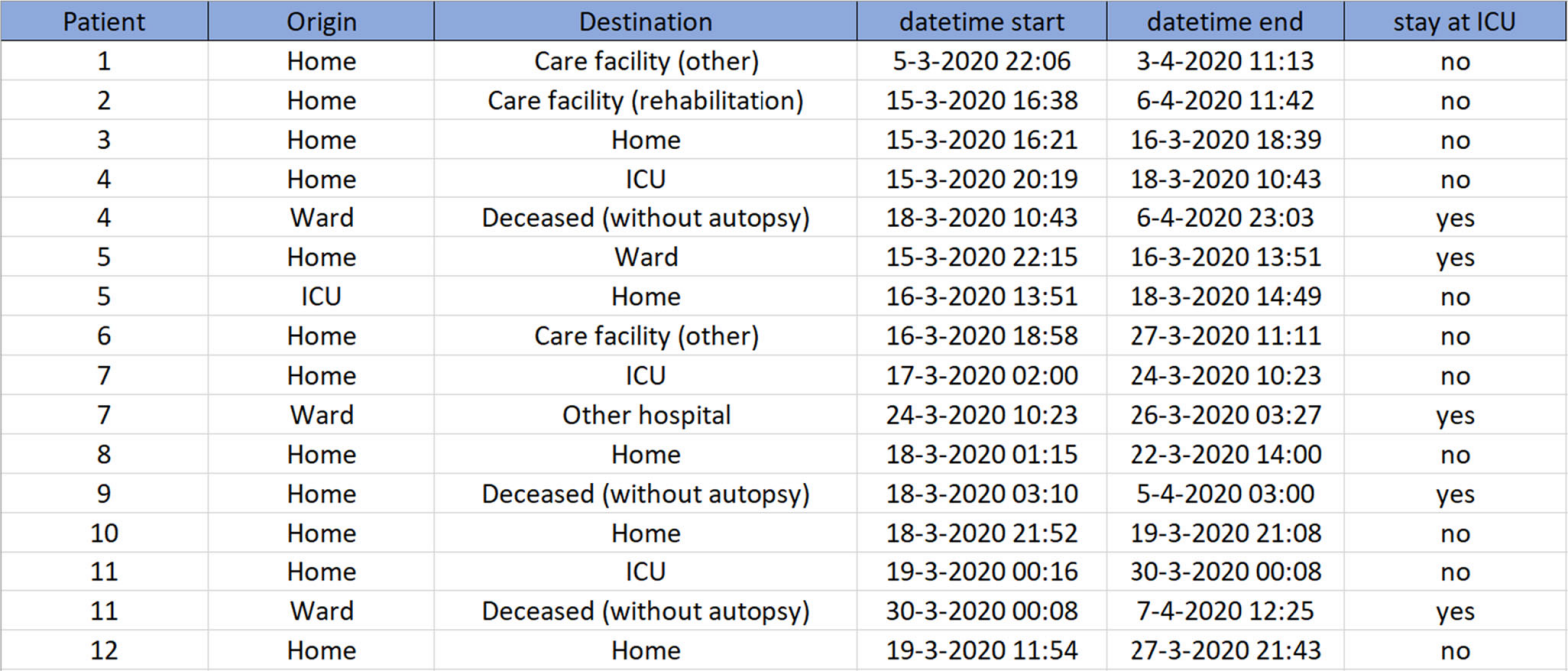
Extract of the input data for our model, where the name of the hospital, patient identification number, as well as patient characteristics such as age, gender and BMI have been removed for privacy reasons. Care facility denotes a long term care facility outside the hospital

Our forecasting method requires a complete record of time stamps for patient admissions, transfers and discharges that we obtained from all hospitals included in this paper. Figure 2 shows an extract of the input table, where the name of the hospital, patient identification number, as well as patient characteristics have been removed for privacy reasons. The rows of this table describe the trajectory of the patient at either the ward or the ICU. The patient ID is replaced by a number in the first column. The next two columns describe the Origin and Destination of the patient before and after his/her stay at the current department, while the last column indicates whether the current department is ICU (yes) or ward (no). The fourth and fifth columns give information about the start and end time of the patient’s stay at the current department. In this table, the trajectory of the patient is explicitly characterised. For example, patient nr. 5 is admitted to the ICU on March 15 at 22:15 from his/her own home environment, transferred to the ward on March 16 at 13:51 and discharged from the ward to return to his/her home environment on March 18 at 14:49.

**Fig. 3:**
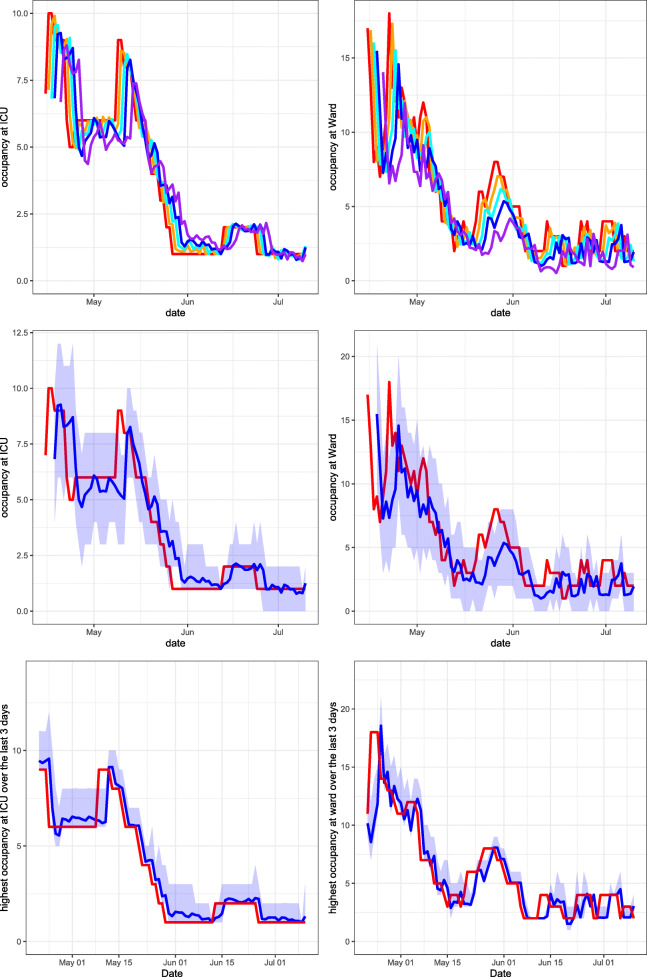
Leiden University Medical Centre April 15, 2020 until July 10, 2020. Top row: expanding window forecasts 1, 2, 3 and 5 days ahead at the COVID-19 ICU (left) and ward (right). Middle row: expanding window forecasts 3 days ahead at the COVID-19 ICU (left) and ward (right), along with a 95*%* prediction interval. Bottom row: expanding window forecasts of the maximum occupancy, including the 95*%* prediction interval and realised maximum occupancy of patients at the COVID-19 ICU (left) and ward (right) over the last 3 days. The realised occupancy is shown in red, while the forecasts for 1, 2, 3 and 5 days ahead are shown in orange, cyan, blue and purple respectively

**Fig. 4:**
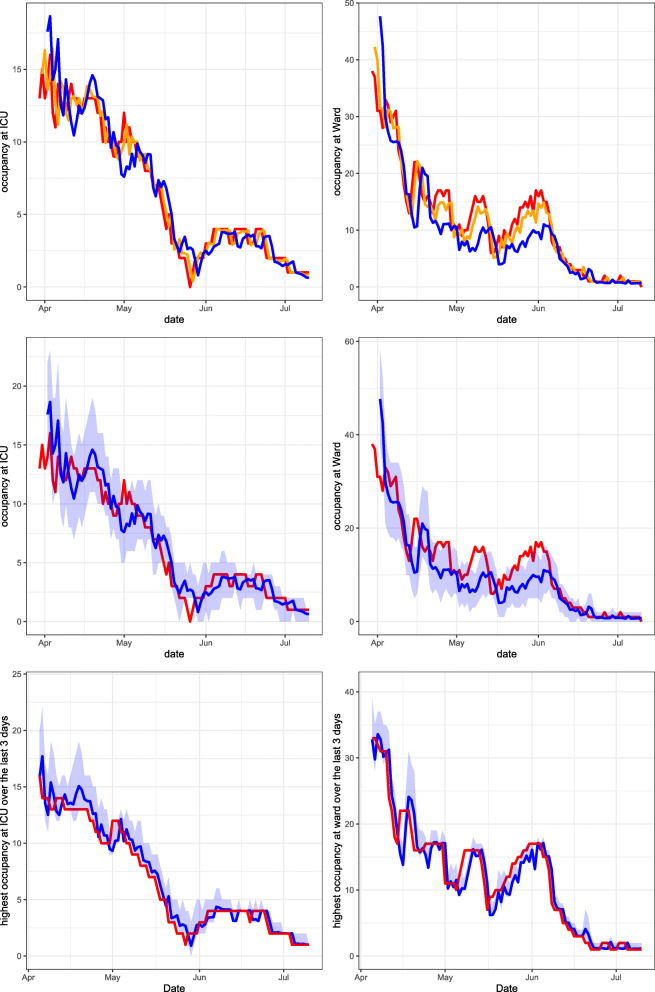
HagaZiekenhuis March 30, 2020 until July 10, 2020. Top row: expanding window forecasts 1 and 3 days ahead at the COVID-19 ICU (left) and ward (right). Middle row: expanding window forecasts 3 days ahead at the COVID-19 ICU (left) and ward (right), along with a 95*%* prediction interval. Bottom row: expanding window forecasts of the maximum occupancy, including the 95*%* prediction interval and realised maximum occupancy of patients at the COVID-19 ICU (left) and ward (right) over the last 3 days. The realised occupancy is shown in red, while the forecasts for 1 and 3 days ahead are shown in orange and blue respectively

**Fig. 5:**
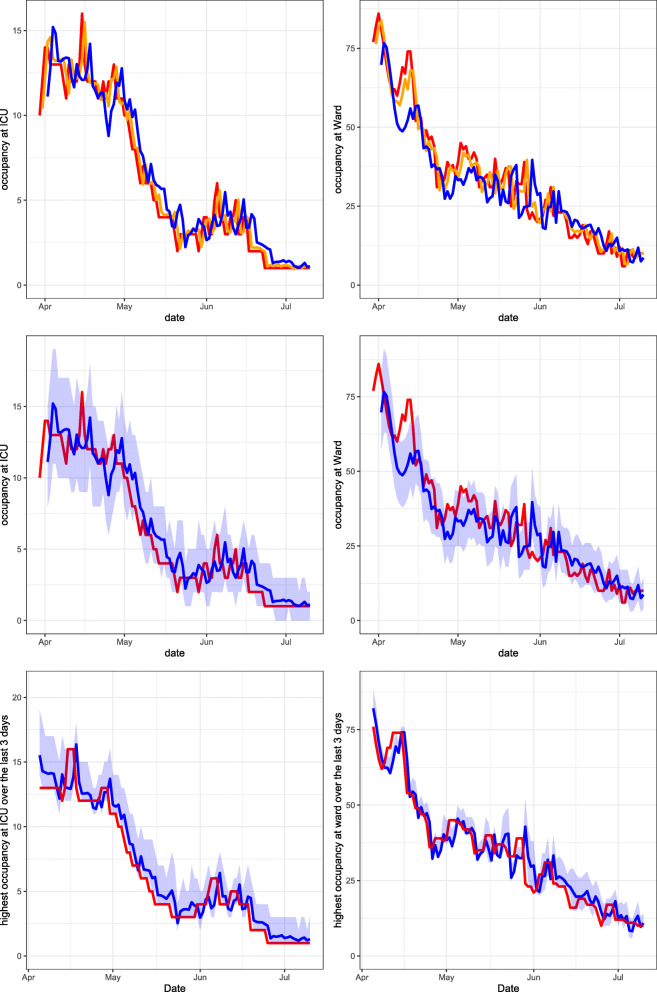
Rijnstate March 30, 2020 until July 10, 2020. Top row: expanding window forecasts 1 and 3 days ahead at the COVID-19 ICU (left) and ward (right). Middle row: expanding window forecasts 3 days ahead at the COVID-19 ICU (left) and ward (right), along with a 95*%* prediction interval. Bottom row: expanding window forecasts of the maximum occupancy, including the 95*%* prediction interval and realised maximum occupancy of patients at the COVID-19 ICU (left) and ward (right) over the last 3 days. The realised occupancy is shown in red, while the forecasts for 1 and 3 days ahead are shown in orange and blue respectively

**Fig. 6:**
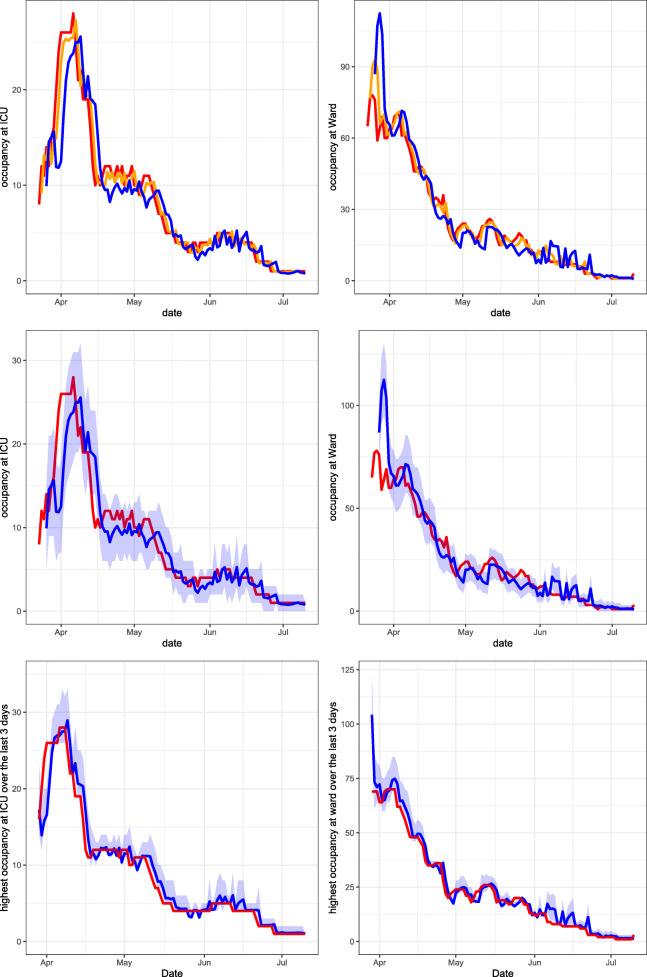
Elisabeth-TweeSteden Ziekenhuis March 23, 2020 until July 10, 2020. Top row: expanding window forecasts 1 and 3 days ahead at the COVID-19 ICU (left) and ward (right). Middle row: expanding window forecasts 3 days ahead at the COVID-19 ICU (left) and ward (right), along with a 95*%* prediction interval. Bottom row: expanding window forecasts of the maximum occupancy, including the 95*%* prediction interval and realised maximum occupancy of patients at the COVID-19 ICU (left) and ward (right) over the last 3 days. The realised occupancy is shown in red, while the forecasts for 1 and 3 days ahead are shown in orange and blue respectively

Using the method described in Section 3.3, the PALM is simulated 1,000 times. In Figs. 3, 4, 5 and 6, performance measures of the true occupancy (at the start of each day, depicted in red) are compared with these measures forecast by our method. Figures 3–6 report estimates of the following performance measures:

1. *Forecasts of the daily bed occupancy at the ward and ICU*:
  13 $$ \begin{array}{@{}rcl@{}} &&\hat{\mathbb{E}}[N_{W}(s+t) | \textbf{L}(s)= {\boldsymbol\ell}], \\ && \hat{\mathbb{E}}[N_{I}(s+t) | \textbf{L}(s)= {\boldsymbol\ell}]. \end{array} $$
  These forecasts are obtained by taking the average of the occupancy at time *t* + *s* for both departments over all 1,000 PALM simulations, given all information **L**(*s*) on the LoSs up to time *s*.
  A forecast of the daily bed occupancy can be used by the hospital to obtain insight in the expected occupancy at both COVID-19 ward and ICU at any given day. Furthermore, when considering multiple forecast horizons *t*, the forecasts give insight into the expected evolution of the occupancy over time.
2. *A 95% prediction interval for the daily occupancy at ward and ICU.* This is the estimator of the expressions in (8) for *α* = 0.95. The quantiles are estimated by calculating the empirical quantiles for level 97.5*%* and 2.5*%* of the occupancy at both departments at time *t* + *s* over all 1,000 PALM simulations.
  This performance measure gives insight into how much capacity is needed for COVID-19 patients on a given day *s* + *t* at both departments and can hence be used for daily capacity planning. In this paper, the level 95*%* is chosen, which can be modified according to the hospital’s risk preferences.
3. *Forecasts of the maximum bed occupancy at the ward and ICU within the forecasting period*:
  14 $$ \begin{array}{@{}rcl@{}} && \hat{\mathbb{E}}\left[\max\limits_{u\in[s,s+t]}N_{W}(u) | \textbf{L}(s)= {\boldsymbol\ell}\right], \\ &&\hat{\mathbb{E}}\left[\max\limits_{u\in[s,s+t]}N_{I}(u) | \textbf{L}(s)= {\boldsymbol\ell}\right]. \end{array} $$
  These forecasts are obtained by taking the average of the maximum occupancy realised for all 1,000 PALM simulations in the forecasting period [*s*,*s* + *t*] for both departments.
  The expected maximum occupancy in a certain forecasting period [*s*,*s* + *t*] expresses the risk of overcrowding of the ward and ICU in the next *t* days. These forecasts can be used to forecast the required number of available beds in the coming days. The expected maximum occupancy is an important performance measure to control admittance of COVID-19 patients to a hospital at both ward and ICU.
4. *A 95% prediction interval of the maximum bed occupancy at the ward and ICU within the forecasting period*. This is the estimator of the expressions in (11) where *α* = 0.95. The quantiles are estimated by calculating the empirical quantiles for level 2.5*%* and 97.5*%* of the realised maximum occupancy at both departments in the forecasting period over all 1,000 PALM simulations.
  Similar to the expected maximum occupancy, the prediction interval for the maximum occupancy gives insight into the risk of overcrowding the ward and ICU. It can be used to control admittance or plan capacity at both departments during the forecasting period. In this paper, the level 95*%* is chosen, which can be modified according to the hospital’s risk preferences.

In the following sections, six graphs are shown for each hospital. The left-hand graphs consider occupancy at the ICU and the right-hand graphs consider occupancy at the ward. For all graphs, the patients transferred from other hospitals are excluded from the calculation of the occupancy. This is because the predicted arrival rate only pertains to autonomous arrivals to the hospital. The forecasts are obtained using an expanding window procedure. This means that for each day, forecasts are generated using only the available data (e.g. arrivals, transfers and departures) up to that day. After calculating the performance measures given all information up to day *s*, the forecast day is incremented by one day, i.e., $s \rightarrow s+1$ and new forecasts are generated using all data up to that new day. This procedure is continued until the end of the pre-specified range of *s* is reached.

In the graphs in the top row, expanding window forecasts of the daily occupancy are shown for each day *s*. Furthermore, for each day *s*, the forecast at day *s* − *t* of the occupancy at day *s* is shown for forecast horizons *t*. The realised occupancy at those days is shown in red. In the top row, the forecasts are shown without prediction intervals. For the LUMC, forecasts for horizons *t* = 1,2,3 and 5 days are shown. For the other hospitals, forecasts for the horizons *t* = 1 and 3 are shown only, to enhance the clarity of the graphs. The colours for forecast horizon *t* = 1,2,3 and 5 are orange, cyan, blue and purple, respectively. The graphs in the middle row display the expanding window forecasts for daily occupancy at forecast horizon *t* = 3 including the 95*%* prediction interval. The graphs in the bottom row show the expanding window forecasts of the expected maximum occupancy, including the 95*%* prediction interval for a forecast horizon of *t* = 3 days.

### Leiden University Medical Centre

Leiden University Medical Centre (LUMC) is an academic hospital in Leiden. Together with the other general hospitals in the region, it serves a community of around two million people in an urban area in the south-west of the Netherlands. The main focus of the LUMC is top clinical and highly specialised care. It is the smallest and oldest of the eight academic hospitals in the Netherlands.

Figure 3 presents our forecasts for the LUMC for the period April 15, 2020 until July 10, 2020, the second part of the first COVID-19 peak in this region. The first COVID-19 patient arrived at the LUMC on March 3, 2020. Hence at the start of the forecast interval, 1.5 months of data is available on the arrival rates and LoSs of COVID-19 patients, including 99 (30) COVID-19 patients that had left the ward (ICU) before then.

### HagaZiekenhuis

HagaZiekenhuis (Haga) is a top clinical hospital in The Hague with 600 beds and approximately 29,000 inpatient admissions per year. Next to secondary care, top clinical hospitals in the Netherlands also provide tertiary care for particular patient groups that the hospital specialises in. Moreover, top clinical hospitals play an important role in the education of medical professionals, and perform clinical research. Haga is located in the same urban area in the south-west of the Netherlands as the LUMC.

Figure 4 presents our forecasts for Haga for the period March 30, 2020 until July 10, 2020, the second part of the first COVID-19 peak in this region. The first COVID-19 patient arrived at Haga on March 5, 2020. Hence at the start of the forecast interval, 25 days of data is available on the arrival rates and LoSs of COVID-19 patients, including 40 (5) COVID-19 patients that had left the ward (ICU) before then.

### Rijnstate

Rijnstate is a top clinical hospital in Arnhem with 766 beds and approximately 33,000 inpatient admissions per year. Rijnstate serves a community of 450 thousand people in and around Arnhem, a city in the east of the Netherlands.

Figure 5 presents our forecasts for Rijnstate for the period March 30, 2020 until July 10, 2020, the second part of the first COVID-19 peak in this region. The first COVID-19 patient arrived at Rijnstate on March 3, 2020. Hence at the start of the forecast interval, approximately one month of data is available on the arrival rates and LoSs of COVID-19 patients, including 157 (7) COVID-19 patients that had left the ward (ICU) before then.

### Elisabeth-TweeSteden Ziekenhuis

Elisabeth-TweeSteden Ziekenhuis (ETZ) is a top clinical hospital in Tilburg with 782 beds and approximately 37,000 inpatient admissions per year. Tilburg is a city in North-Brabant, the province that experienced the initial outbreak of COVID-19 in the Netherlands at the end of February, 2020. As a consequence, ETZ was the first Dutch hospital to admit a COVID-19 patient. Just like HagaZiekenhuis and Rijnstate, ETZ is a top clinical hospital.

Figure 6 presents our forecasts for ETZ for the period March 23, 2020 until July 10, 2020, the second part of the first COVID-19 peak in this region. The first COVID-19 patient arrived at ETZ on February 28, 2020. Hence at the start of the forecast interval, almost one month of data is available on the arrival rates and LoSs of COVID-19 patients, including 104 (24) COVID-19 patients that had left the ward (ICU) before then.

### Evaluation of our method

In this section, we discuss the results presented in Sections 4.1–4.4. Moreover, we compare the performance of our forecasting method to the performance of a moving average forecaster for all four hospitals.

When investigating trends in the COVID-19 occupancy during the first peak, we see a very similar trend for the LUMC (Fig. 3) and Haga (Fig. 4), which is natural as these two hospital are located in the same region. ETZ (Fig. 6) has the earliest and highest peak and also admitted the highest total number of COVID-19 patients, because ETZ is located in North-Brabant, the region that was hit first and hardest during the first COVID-19 peak in the Netherlands. In Rijnstate (Fig. 5) the first COVID-19 peak ended the latest.

Naturally, the accuracy of forecasts increases when forecasts are made at a point in time that is closer to the actual realisation. This can clearly be seen in the top rows of Figs. 3–6. In particular, the top row of Fig. 3 shows that our expanding window forecasts 1 day ahead are very accurate, while the accuracy reduces for forecasts 5 days ahead. One of the reasons is that the 1-day forecast is able to pick up sudden changes in the trend the next day, whereas this obviously takes five days for the 5-day forecast. This is, for example, visible in the top-right graph of Fig. 3 when, mid-May, the downward trend changes to a sudden peak. Similar effects are visible in the same graph end-May and also in Figs. 4–6 with a sudden decline in the number of hospitalised patients. This delay in picking up sudden changes in the trend also results in larger over- or undershoots for the forecasts further into the future.

The top and middle rows of Figs. 3–6 also show clearly that the accuracy of forecasts increases for larger population sizes. ETZ has seen the highest number of COVID-19 patients on its ward and ICU during the first peak, and indeed the middle row of Fig. 6 displays narrow confidence intervals that often contain the realisation. LUMC (Fig. 3) and Rijnstate (Fig. 5) saw the smallest number of COVID-19 ward and ICU patients, respectively, resulting in broader confidence intervals that contain the realisation less often.

In contrast to the forecasts of the daily occupancy that we just discussed, the forecast of the maximum occupancy 3 days ahead of time is very close to the realisation, as displayed in the bottom rows of Figs. 3–6. This is exactly the forecast that is most valuable for hospitals, as it provides quantitative support for several decisions, for example on the admittance of additional COVID-19 patients, the necessity of COVID-19 patient transfers to other hospitals, and the (im)possibility of providing care for non-COVID-19 patients.

Table 1 displays error measures for our forecasting method and also for a moving average forecaster, which enables us to compare the results. For the moving average forecaster, the average occupancy over the last week is used as forecast of the daily occupancy for several horizons *t*. Error measures for the expected maximum bed occupancy are not displayed, as it is not straightforward to forecast the maximum occupancy using a moving average.

**Table 1:** Results for our forecasting method (average over 10 simulation runs of 1,000 replications per hospital) compared to a moving average forecaster for the occupancy at the departments of the hospitals

	Forecast						Mov. av. forecaster			
	ICU			Ward			ICU		Ward	
	CR	bias	MAE	CR	bias	MAE	bias	MAE	bias	MAE
LUMC										
1 day ah.	0.96	0.07	0.30	0.57	− 0.34	1.04	0.41	0.73	0.37	1.52
2 days ah.	0.94	0.10	0.51	0.64	− 0.69	1.29	0.47	0.86	0.58	1.67
3 days ah.	0.90	0.12	0.67	0.67	− 0.99	1.66	0.53	0.98	0.71	1.85
5 days ah.	0.95	0.11	0.91	0.65	− 1.47	1.96	0.66	1.22	0.98	2.14
Max. 3d. ah.	0.84	0.26	0.53	0.21	− 0.35	1.33	–	–	–	–
Haga										
1 day ah.	0.93	0.13	0.62	0.76	− 0.76	1.54	0.50	0.94	1.29	2.68
2 days ah.	0.94	0.16	0.89	0.75	− 1.54	2.43	0.65	1.11	1.60	3.05
3 days ah.	0.94	0.15	1.03	0.66	− 2.23	3.09	0.78	1.26	1.93	3.46
5 days ah.	0.94	0.21	1.41	0.58	− 3.03	4.25	1.03	1.52	2.54	4.26
Max. 3d. ah.	0.78	0.31	0.76	0.19	− 0.18	1.69	–	–	–	–
Rijnstate										
1 day ah.	0.98	0.19	0.59	0.80	− 0.27	3.58	0.50	0.85	2.68	4.75
2 days ah.	0.97	0.32	0.84	0.77	− 1.05	4.78	0.63	1.00	3.26	5.21
3 days ah.	0.99	0.42	0.96	0.70	− 1.68	5.79	0.77	1.13	3.85	5.69
5 days ah.	0.97	0.55	1.18	0.72	− 3.30	6.93	1.01	1.36	5.09	6.55
Max. 3d. ah.	0.81	0.45	0.94	0.40	0.48	4.00	–	–	–	–
ETZ										
1 day ah.	0.97	− 0.13	0.88	0.84	0.22	1.77	0.49	1.70	2.60	3.56
2 days ah.	0.94	− 0.28	1.29	0.81	0.43	3.07	0.68	1.99	3.21	4.19
3 days ah.	0.94	− 0.44	1.69	0.78	0.75	4.34	0.92	2.25	3.82	4.72
5 days ah.	0.90	− 0.82	2.39	0.72	0.65	6.10	1.44	2.65	5.17	5.73
Max. 3d. ah.	0.78	0.24	1.13	0.28	1.74	2.84	–	–	–	–

CR: coverage rate of the occupancy by the 95% prediction interval, bias: the bias estimated by averaging errors, MAE: mean absolute error

To increase the reproducibility of the comparison, the results for our forecasting method are averaged over 10 evaluations, i.e., 10 repetitions of the expanding window procedure. The maximal standard deviation in the results, taken over all departments and hospitals, was 0.02, which shows that there is little variation over different evaluations. Using a 95*%* two sided confidence interval based on Student’s t-distribution (df = 9), it holds that a difference in error values between the moving average forecaster and our forecast method exceeding $1.833\cdot 0.02/\sqrt {10}\approx 0.012$ is statistically significant with respect to the noise over different evaluations.

The columns ‘CR’ (coverage rate) indicate how often the realised bed occupancy was covered by the 95% prediction interval. For the ICU, this happened in 78 – 99% of cases. The lower coverage rate at the ward, resulting from narrow prediction intervals, is most likely due to the fact that we do not incorporate the uncertainty about the estimated LoS distributions and predicted arrival rates in our prediction intervals, which would widen these intervals resulting in increased coverage rate. Most of the occupancy in the ward is due to direct arrivals to the hospital, while the uncertainty was seen to be highest for the arrival rate predictions. Observe from Figs. 3–6 that when the prediction interval does not cover the realisation, it is mostly very close to the realisation. The bias is calculated as the average of ‘forecast minus realised’. Hence, the closer to zero the better. For the ICU, the absolute bias of our forecast is always lower than 1, and lower than the bias of the moving average forecaster. For the ward, the negative bias indicates that our forecast is slightly too low on average. For the wards at ETZ and Rijnstate, which admitted the highest numbers of patients, the bias of our forecast is much lower than that of the moving average forecaster. The mean absolute error (MAE) of our forecast is again close to zero for the ICU, and lower than that of the moving average forecaster for all hospitals. For the ward, the MAE of our forecast is lower than that of the moving average forecaster, except for horizons 3,5 at Rijnstate and horizon 5 at ETZ. In conclusion, our forecasting method often outperforms the moving average forecaster. Moreover, our forecasting method is richer as it also produces prediction intervals and generates forecasts for the maximum occupancy that are very close to the realisation.

**Fig. 7:**
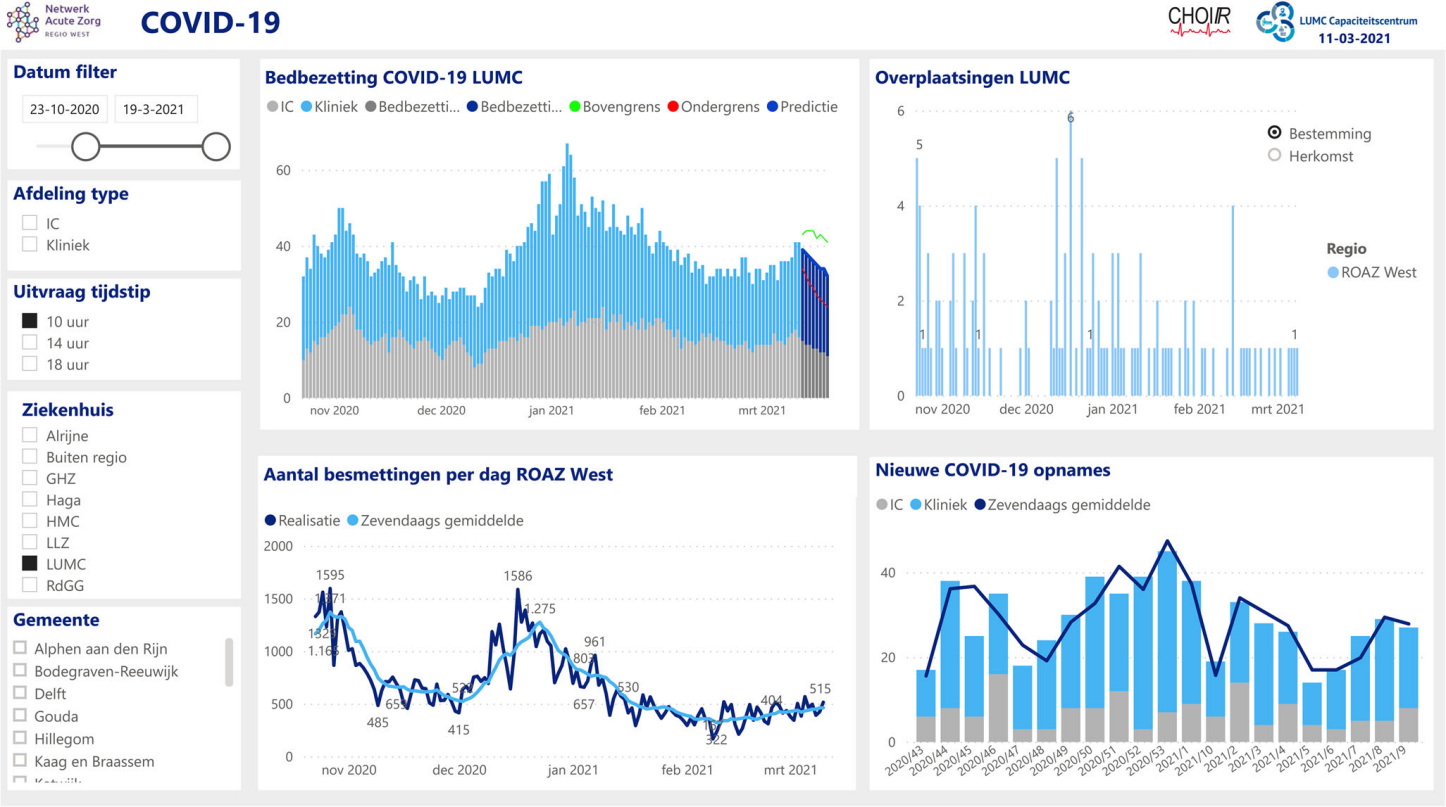
The LUMC capacity dashboard, with our forecasts of the bed occupancy incorporated in the darker (right) part of the top-left graph provided with the prediction interval (green and red)

To summarise, our forecasting method shows to be very accurate, which has convinced hospitals to embrace our forecasting method and incorporate it in their COVID-19 control or capacity dashboard (see Fig. 7).

## Discussion and conclusion

In this paper, we have presented a data-driven approach that forecasts the number of hospitalised COVID-19 patients in the ward and the ICU based on predicted arrival rates and estimated LoSs, justified by an underlying network of infinite server queues driven by a Poisson Arrival Location Model (PALM). As demonstrated in Section 4, that reports the results of our method for the first COVID-19 peak, the forecasts produced by our method are very accurate. In particular, the forecasts of the maximum occupancy in the ward and the ICU three days ahead are very close to the realisation. This enables hospitals to make informed decisions about whether or not to admit additional COVID-19 patients at their ward or ICU. Indeed, our forecasts are currently being used in four Dutch hospitals during the second COVID-19 peak the Netherlands is facing. For example, the LUMC has incorporated our forecasting graphs in their capacity dashboard (see Fig. 7), which is now being reviewed on a daily basis by their physicians.

We have modelled the ward and ICU as a network of infinite server queues. Clearly, the number of beds at the ward and ICU are finite so that a network of Erlang loss queues seems a more natural model. However, as we are interested in forecasting the risk of bed shortage due to autonomous patient arrivals, expressed as the probability that the maximum number of COVID-19 patients in the ward and ICU exceeds a pre-specified safety level, a network of infinite server queues without capacity restrictions on the maximum number of patients present in the ward and ICU is more natural. In addition, also for the forecast of the number of patients present in the ward and ICU the network of infinite server queues yields a good approximation of the network of Erlang loss queues, as supported by literature and by the accuracy of our forecasts.

The daily number of autonomous arrivals is predicted using a Richards’ curve estimated using the Levenberg-Marquardt algorithm. Just as in [31], we observed that for early stage data, the algorithm suffers from instability due to the fact that some of the characteristics of the curve cannot be estimated well for such data sets. The proposed solution in [31] is to forecast very early stage arrivals using an exponential curve, and to forecast the other early stage arrivals using the logistic curve. For all of the scenarios evaluated in this paper, the algorithm was able to estimate parameters for either the Richards’ or the logistic curve, hence exponential extrapolation was not implemented.

Our model takes into account patient characteristics. However, in practice, patient characteristics are often not available or only available for a subset of patients. Therefore, in our generation of the PALM, the patient characteristics only describe the paths these patients will follow through the system, and hence do not take further patient characteristics and entrance times into account. Despite the fact that we do not incorporate further patient characteristics such as age, gender, and BMI, our results yield accurate forecasts of the number of patients in the ward and ICU. Additional patient characteristics are included in our model. The impact of additional patient characteristics on the LoS and transfer probabilities is an interesting topic for future research.

**Fig. 8:**
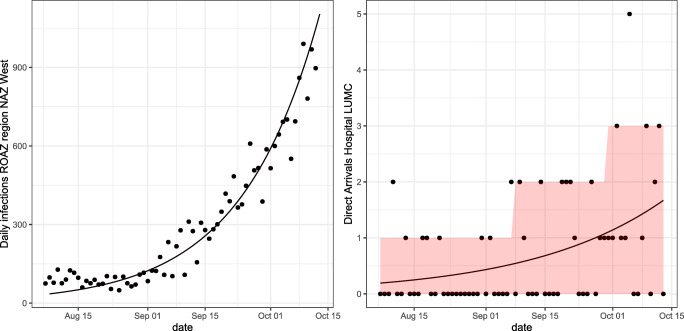
Left: the number of daily infections in ROAZ region NAZ West and the trend given by our prediction method using the Richards’ curve fitted on the (cumulative) daily number of infections. Right: the daily number of autonomous direct arrivals to the LUMC. The trend and 80*%* prediction interval given by our forecasting method using the Richards’ curve fitted on this arrival data is also shown for the LUMC. Both plots are shown for the period starting from 8-07-2020 until 14-10-2020

Now that we have developed a forecasting method that enables informed decision-making for individual hospitals, in future research we aim to build on this method to develop a regional model. Our regional model will not only forecast the COVID-19 occupancy in several hospitals, but also use these forecasts to provide decision support for proactively transferring COVID-19 patients from one hospital in the region to another when the first faces a risk of overcrowding. In our regional model, we will apply a Richards’ curve to predict the daily regional number of COVID-19 patients that require hospitalisation, instead of autonomous direct arrivals to each of the hospitals, as this provides an accurate approximation at the regional level. Figure 8 shows the Richards’ curve for the LUMC and for the region NAZ West that contains the LUMC. As we aim for a model that provides decision support for patient transfers between hospitals in the region, interaction between the hospitals clearly plays a major role. Therefore, we plan to combine our current research with earlier work on managing the overflow of ICU patients within a region [17], where we may invoke the Modified Offered Load approximation [1, 19] to take into account the capacity constraints on the number of available beds in the hospitals.

Given the quality of our forecasts and the swift implementation of our forecasting method in four Dutch hospitals, we are confident that hospitals will also embrace our regional model. As such, the outlook is that we can provide decision support for one of the major COVID-19 challenges in the Netherlands – transferring COVID-19 patients between hospitals.

More information on the forecasting method, access to the code and related research can be found on: www.utwente.nl/en/choir/research/Covid19-wardICU/.
